# Functional expression and purification of DoxA, a key cytochrome P450 from *Streptomyces peucetius* ATCC 27952

**DOI:** 10.7717/peerj.14373

**Published:** 2022-11-16

**Authors:** Liyan Yang, Dengfeng Yang, Qingyan Wang, Juan Li, Hong-Liang Li, Lixia Pan

**Affiliations:** 1 National Engineering Research Center for Non-Food Biorefinery, State Key Laboratory of Non-Food Biomass and Enzyme Technology, Guangxi Academy of Sciences, Nanning, China; 2 Guangxi Key Laboratory of Marine Natural Products and Combinatorial Biosynthesis Chemistry, Guangxi Academy of Sciences, Nanning, China

**Keywords:** Cytochrome P450, DoxA, Doxorubicin synthase, Functional expression, Purification

## Abstract

The antitumor drug doxorubicin is widely used in clinical practice. However, the low yield and high cost of this drug highlight the urgent need for cost-effective processes to rapidly manufacture antitumor drugs at scale. In the biosynthesis pathway, the multi-functional cytochrome P450 enzyme DoxA catalyzes the last three steps of hydroxylation. The final conversion of daunorubicin to doxorubicin is the rate-limiting step. In our work, the DoxA has been expressed with the ferredoxin reductase FDR2 and the ferredoxin FDX1 and purified to homogeneous. The reduced carbon monoxide difference spectroscopy, heme concentration, and enzymatic characteristic were characterized. These studies suggest an approach for engineering *Streptomyces* P450s with functional expression for mechanistic and structural studies.

## Introduction

Cytochrome P450 (P450) is a ubiquitous heme-dependent enzymes that catalyze multiple reactions through a complex multistep mechanism (Rudolf et al., 2017). As the prosthetic group of P450 enzymes, heme is linked to an absolutely conserved cysteine. P450 was named as its reduced state produces a characteristic absorption peak at 450 nm when combined with carbon monoxide (Klingenberg, 1958; Omura & Sato, 1962). The P450s typically act as monooxygenases, binding dioxygen to their ferrous heme iron and ultimately inserting an atom of oxygen into the substrate, with the other oxygen atom being reduced to water (Rudolf et al., 2017). They are well known for their roles in human heterologous detoxification, steroid biosynthesis and drug metabolism (de Montellano, 2015), but also play a key role in the biosynthesis of natural products (Podust & Sherman, 2012; de Montellano, 2015). One key structural feature of P450s is the coordination of the thiolate anion of cysteine to the heme iron as the fifth ligand in the active form (Sono, Andersson & Dawson, 1982; Champion et al., 1982; Stern & Peisach, 1974; Collman & Sorrell, 1975; Sun et al., 2013). The biologically inactive conformation of a cytochrome P450 protein is usually denoted as the P420 form, which is characterized by a CO bound Soret peak at 420 nm (Sun et al., 2013). *Streptomyces* P450s are expressed sometimes as P420 forms, the biologically inactive forms which possess ferrous CO Soret absorption at 420 nm (Healy et al., 2002). Therefore, it is crucial to obtain high-level expression and active P450 for its function research.

**Figure 1:**
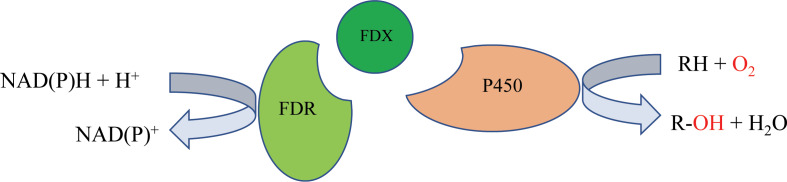
The mechanism of bacterial type I P450-related electron-transport system.

P450 systems mainly comprise two functional parts: the heme-containing P450 domain, and redox partners (RPs). The heme-containing P450 domain contributes to the binding and transformation of substrate, and the RPs contain redox centers to relay electron equivalents from electron donors, NAD(P)H in most cases, to activate the dioxygen bound to the P450 domain (Chen et al., 2021). P450 systems can be classified based on the redox partners required for catalytic activity. In general, a P450 catalytic system includes four components: the substrate, a P450 enzyme for substrate binding and oxidative catalysis, the redox partner(s) that functions as an electron transfer shuttle, and the cofactor, which provides the reducing equivalents (Li et al., 2020). Most bacterial P450s belong to the Class I P450 system, which require two RP proteins: an NAD(P)H-dependent ferredoxin reductase (FdR) and ferredoxin (Fdx), and the electron transfer chain is NAD(P)H →FAD →Fe-S cluster →heme (Hannemann et al., 2007) (Fig. 1).

**Figure 2:**
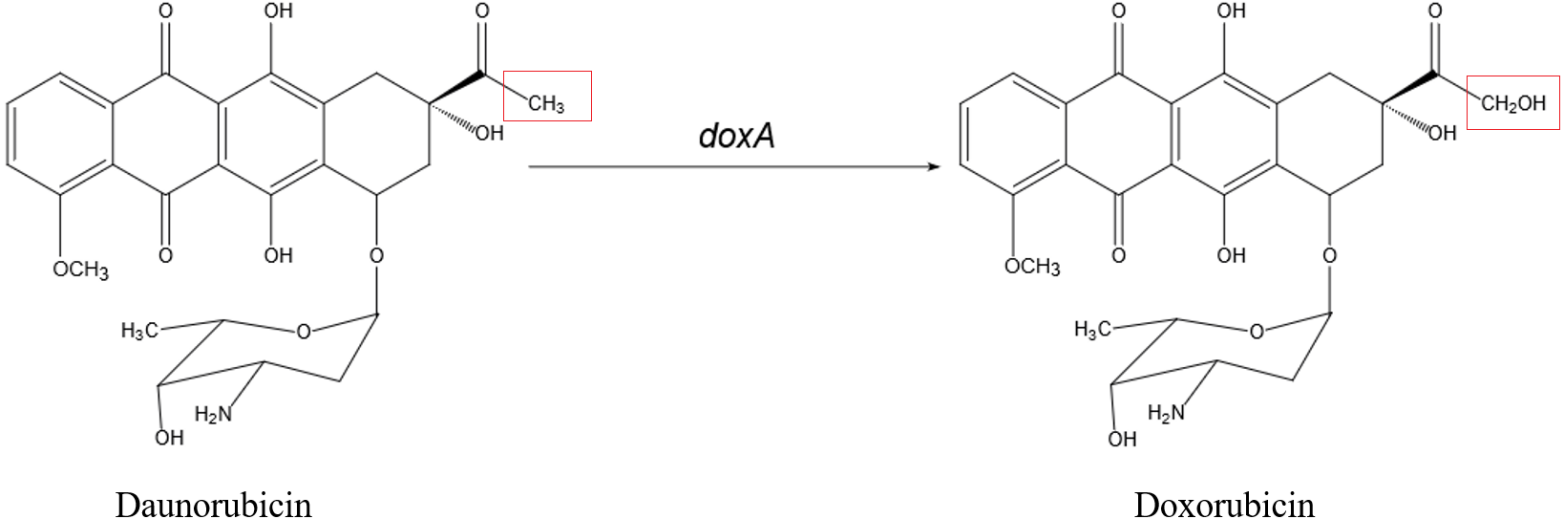
The biosynthetic pathway catalyzed by DoxA. The red box is the difference between daunorubicin and doxorubicin.

Doxorubicin (DXR) is a potent antitumor drug which is the anthracycline drug. In the biosynthesis of DXR, multi-functional Cytochrome P450 enzyme DoxA is responsible for the final three-step hydroxylation (Fig. 2). DoxA has been purified by Rimal et al. (2015) but it is mostly in inactive P420 forms (Fig. S1). In order to acquire more active DoxA to study its function and structure, we tried different constructions to express DoxA in this paper. There are six FDXs and seven FDRs in *Streptomyces peucetius*, and the redox partner of DoxA has been identified by Rimal et al. (2015), their study suggested the primary electron-transport pathway of DoxA is NADH →FDR2 →FDX1 →DoxA. Most P450s require redox partner proteins to sequentially transfer two electrons form NAD(P)H to their heme–iron reactive center for dioxygen activation (Ruettinger & Fulco, 1981). In this study, FDR2, FDX1 and DoxA of *Streptomyces peucetius* were co-expressed in *E. coli* expression system. The unprecedented high-efficiency and functional expression and purification of DoxA in *E. coli* expression system was realized, and the enzymatic assay of DoxA using daunorubicin (DNR) as the substrate was also performed.

## Material and Methods

### Strains and materials

*E. coli* strains were grown at 37 °C in Luria Bertani (LB) media in both liquid and agar plates supplemented with the appropriate amount of antibiotic. *E. coli* DH5 *α* was used for recombinant plasmid construction. *E. coli* BL21 Codon plus (DE3) RIL was used as protein expression host. The plasmids pET22b, pET28a, pRSFDuet and pETDuet (Table 1) were used as expression vectors. Antibiotics were added at the following concentrations for *E. coli*: kanamycin (Kan) 50 µg/ml; ampicillin (Amp) 100 µg/ml; chloramphenicol (Cm) 25 µg/ml. The following supplement was added when required: isopropyl- *β*-D-thiogalactopyranoside (IPTG) 0.1 mM; Fe^2+^ 0.5 mM, *σ*-aminol evulinic acid (ALA) 0.5 mM.

**Table 1:** Bacterial strains and plasmids used in this work.

Strains or plasmids	Relevant characteristics^a^	Reference or source
*Escherichia coli*		
DH5 *α*	F^−^Φ80*lac* Z ΔM15 Δ(*lac* ZYA-*arg* F) U169 *rec* A1 *end* A1 *hsd* R17(r_k_^−^,m${}_{\mathrm{k}}^{+}$) *pho* A *sup* E44 *thi*-1 *gyr* A96 *rel* A1 *λ*^−^	Gibco BRL, Life Technologies
BL21 Codon plus (DE3) RIL	F^−^*ompThsdS* (r${}_{\mathrm{B}}^{-}$ m${}_{\mathrm{B}}^{--}$) *dcm*^+^*galλ*(DE3) *end* A Hte [*argU ileY leuW* Cam^r^ ]	Novagen
22bA/RIL	BL21 Codon plus (DE3) RIL harboring 22bA, Amp^r^, Cam^r^	This work
28Aa/RIL	BL21 Codon plus (DE3) RIL harboring 28aA, Kan^r^, Cam^r^	This work
pEA/RIL	BL21 Codon plus (DE3) RIL harboring pEA, Kan^r^, Cam^r^	This work
pEAX1/RIL	BL21 Codon plus (DE3) RIL harboring pEAX1, Kan^r^, Cam^r^	This work
AX1R2/RIL	BL21 Codon plus (DE3) RIL harboring pEAX1 and pER2, Kan^r^, Amp^r^, Cam^r^	This work
Plasmids		
pET22b	Expression vector, C-terminal 6 ×His-tagged sequences, Amp^r^	Novagen
pET28a	Expression vector, N-terminal 6 ×His-tagged sequences, Kan^r^	Novagen
pRSFDuet	Expression vector which contains two multiple cloning sites (MCS), N-terminal 6 ×His-tagged sequences, Kan^r^	Novagen
pETDuet	Expression vector which contains two multiple cloning sites (MCS), N-terminal 6 ×His-tagged sequences, Amp^r^	Novagen
22bA	pET22b containing *doxA* coding region, Amp^r^	This work
28aA	pET28a containing *doxA* coding region, Kan^r^	This work
pEA	pRSFDuet containing *doxA* coding region, Kan^r^	This work
pEAX1	pRSFDuet containing *doxA* and *fdx1* coding region, Kan^r^	This work
pER2	pETDuet containing *fdr2* coding region, Amp^r^	This work

**Notes.**

a Kan^r^, Amp^r^ and Cam^r^ indicate resistance to kanamycin, ampicillin and chloramphenicol, respectively.

### Molecular cloning and construction of recombinant plasmids

*doxA* gene for construction to different expression vector was amplified with primers listed in Table 2. Ferredoxin (*fdx1*) gene and Ferredoxin reductase (*fdr2*) gene were amplified with primer sets fdx1-F/R and fdr2-F/R (Table 2), respectively. Total DNA of *S. peucetius* 27952 strain was used as the PCR template. PCR products were purified with PCR clean-up kit according to manufacturer’s description.

**Table 2:** Primers used in this study.

**Primer Name**	**Sequence**
22b-doxA-F	taagaaggagatatacatatgGTGAGCGGCGAGGCGCCC
22b-doxA-R	gtggtggtggtggtgctcgagGCGCAGCCAGACGGGCAG
28a-doxA-F	gtgccgcgcggcagccatatgGTGAGCGGCGAGGCGCCC
28a-doxA-R	ctcgagtgcggccgcaagcttTCAGCGCAGCCAGACGGG
RSF-doxA-F	ccacagccagggatccgaattcGTGAGCGGCGAGGCGCCC
RSF-doxA-R	gcattatgcggccgcaagcttTCAGCGCAGCCAGACGGG
fdx1-F	taagaaggagatatacatatgATGACCGTGCAGCACGAGG
fdx1-R	ggtttctttaccagactcgagTCACTCCGCGTCCGGGCC
fdr2-F	agatatacatatggcagatctGCATCACCATCATCACCACCTTCGCATCGCCGTC
fdr2-R	ggtttctttaccagactcgagTCAGCGGGCCGCGTCCGG

The purified *doxA* fragment was ligated into pRSFDuet that was digested with *Eco* R I and *Hin* d III by ClonExpress II One Step Cloning Kit (Vazyme, China), generating the recombinant plasmid named pEA (Table 1) and its structure is shown in Fig. S2. Other expression plasmids 22bA and 28aA (Table 1) were constructed by the same strategy. The plasmids pEA, 22bA and 28aA were respectively transformed into expression host BL21 Codon plus (DE3) RIL generating the strains pEA/RIL, 22bA /RIL, and 28aA /RIL.

The purified *fdx1* fragment was ligated into the multiple cloning site (MCS) 2 of pRSFDuet-1 that was digested with *Bgl* II and *Xho* I by ClonExpress II One Step Cloning Kit (Vazyme, China), of the resulting vectors containing *doxA* gene in the MCS 1 and *fdx1* gene in the MCS 2, generating the recombinant plasmid named pEAX1 (Table 2). The plasmid pEAX1 was transformed into BL21 Codon plus (DE3) RIL, generating the strain pEAX1/RIL. The purified *fdr2* fragment was ligated into the MCS 2 of pETDuet that was digested with *Bgl* II and *Xho* I by ClonExpress II One Step Cloning Kit (Vazyme, China), generating the recombinant plasmid named pER2. The plasmid pEAX1 and pER2 were co-transformed into BL21 Codon plus (DE3) RIL, generating the strain AX1R2/RIL.

### Overexpression of *doxA*, *fdx1* and *fdr2* in *E. coli* strains

A single colony of AX1R2/RIL was inoculated in 10 mL LB medium over night at 37 °C as the seed culture. One percent seed were transferred in 500 mL LB medium in 2 L shaking flask. Cells were grown at 37 °C to OD_600_ about 0.6−0.8, then induced by adding IPTG to final concentration of 0.1 mM, ALA and Fe^2+^ were added to 0.5 mM, and the cells were incubated for 24 h at 16 °C. The cell pellets were harvested by centrifugation at 7,000 g for 15 min and resuspended with buffer containing 50 mM Tris, 300 mM NaCl, 20% glycerol, pH 7.5. Finally, the cell pellets were lysed by ultra-sonication. The soluble protein was separated from the cell debris by centrifugation at 12,000 rpm for 30 min at 4 °C. Other strains were expressed by the same strategy.

### Purification and isolation of DoxA, FDX1 and FDR2

After centrifugation, the supernatant was loaded onto a column containing HisPur™ Ni-NTA Resin (GE Healthcare) for His-tag affinity purification. The column was washed five times with wash buffer (50 mM Tris, 300 mM NaCl, 20% glycerol, 50 mM imidazole, pH 7.5) to remove contaminating proteins. The target protein was eluted with elution buffer (50 mM Tris, 300 mM NaCl, 20% glycerol, 500 mM imidazole, pH 7.5). The finally obtained protein was analyzed using 12% sodium dodecyl sulfate polyacrylamide gel electrophoresis (SDS-PAGE).

FDR2 was further purified by size-exclusion chromatography at 10−20 °C on gel filtration column (GE Healthcare, Superdex 75), in three protein loads on a column. Then FDX1 was further purified by ion exchange column (GE Healthcare) with buffer A (20 mM MES, 20% glycerol, pH 6.5) and buffer B (20 mM MES, 1M NaCl, 20% glycerol, pH 6.5), in two proteins loads on a column. Purified fractions were checked on SDS-PAGE gel. Protein concentrations were measured with NanoDrop Spectrophotometer (Thermo Scientific) at 280 nm. Purified proteins were snap-frozen in liquid nitrogen and stored at −80 °C.

### CO-binding CYP assay

The reduced-CO difference spectrum of the DoxA was obtained according to Liu et al. (Liu et al., 2003). Briefly, the purified DoxA protein was reduced with a few sodium dithionites, and the sample was scan between 400 nm and 500 nm at room temperature. Finally, the sample cuvette was saturated with about 30 bubbles to 40 bubbles of CO at a rate of 1 bubble per second, and was scanned between 400 nm and 500 nm at room temperature. The concentration of active P450 was calculated as described by Omura & Sato (1964).

### Pyridine hemochromagen assay for the determination of heme protein concentration

The determination of heme protein concentration was according to Barr and Guo (Barr & Guo, 2015). Solution I which contains 0.2 M NaOH, 40% (v/v) pyridine, 500 µM potassium ferricyanide (K_3_Fe(CN)_6_) was prepared in a tube. The solution of 0.5 M sodium dithionite was prepared in 0.5 M NaOH in another tube. A cuvette containing 500 µL solution I and 500 µL buffer A was used as a reference for all absorbance measurements. 500 µL of purified protein in buffer A and 500 µL of the solution I were transferred to a cuvette and mixed well. The UV–Vis spectrum of the oxidized Fe III state was recorded immediately. To the cuvette was then added 10 µL of the sodium dithionite solution, and the UV–Vis spectrum of the reduced Fe II state was recorded immediately.

### Enzyme assay of DoxA

The activity of DoxA was assayed using the DNR (daunorubicin) substrate. The reaction mixture consisted of 6 mg mixed protein (DoxA+FDX1+FDR2), 50 µM glucose-6-phosphate, 0.5 U glucose-6-phosphate dehydrogenase, 200 µM cysteine, 5 µM NADPH, 5mM MgCl_2_ and 100 µM DNR, and the reaction was carried out in 20 mM sodium phosphate buffer (pH 7.5). The reaction mixtures were incubated at 30 °C for 24 h, and the pH was adjusted to 8.0 to stop the reaction. The solution was subsequently freeze-dried and was diluted with methanol. The product was analyzed using high performance liquid chromatography (HPLC; Waters, Milford, MA, USA). The HPLC was performed under the following condition: Kromasil C18 (250 mm ×4.6 mm I.D.), composed of solvent A [pH 2.3, with TFA (v/v)] in water and solvent B (100% methanol), were used in a flow rate of 1.0 ml min^−1^. Detection was carried out with a UV detector at 254 nm.

## Results

### The expression of DoxA in pET22b and pET28a

In order to express DoxA protein (48 kDa), *doxA* gene was cloned into pET22b which contains C-His_6_-tag and pET28a which contains N-His_6_-tag. The C-His_6_-tagged and N-His_6_-tagged fusion protein DoxA could be expressed well (Fig. S3). However, the amount of protein seen as a band on an SDS-PAGE gel does not show how much of the protein is correctly folded and active (Hussain & Ward, 2003). Therefore, we need to detect whether the DoxA protein folds correctly.

**Figure 3:**
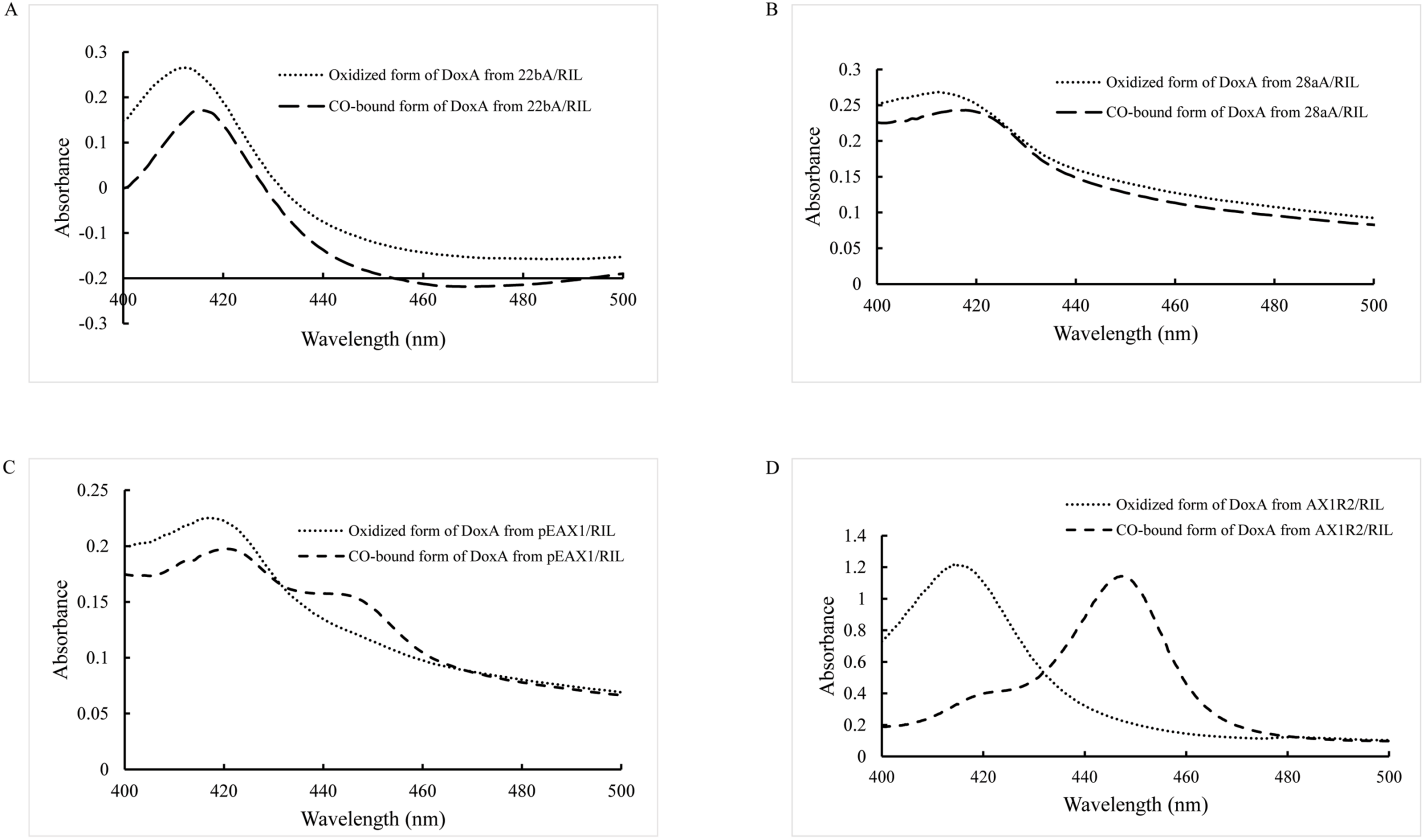
CO-binding spectra of DoxA purified from 22bA/RIL (A), 28aA/RIL (B), pEAX1/RIL (C) and AX1R2/RIL (D).

As a P450 enzyme, the absorption peak of DoxA shifts from 420 nm to 450 nm when CO is added into the reducing agent, which can be used as a characteristic to detect the activity of DoxA. Therefore, evaluation of the levels of correctly folded recombinant P450 was carried out by determining the CO-reduced difference spectra (Haudenschild et al., 2000; Simgen et al., 2000). The purified DoxA was added with reducing agent sodium dithionite and CO was introduced, the absorbing form of DoxA was almost at 420 nm (Figs. 3A, 3B), which suggested DoxA fused with pET22b and pET28a may be folded incorrectly.

### Expression of DoxA in the presence of ferredoxin and ferredoxin reductase

Most CYPs require redox partner proteins to sequentially transfer two electrons form NAD(P)H to their heme–iron reactive center for dioxygen activation (Sun et al., 2017). The activity of correctly folded cytochrome P450s was further enhanced by cloning a ferredoxin reductase (Hussain & Ward, 2003). The study by Rimal et al. (2015) showed the most appropriate redox partners of DoxA are ferredoxin FDX1 (18 kDa) and ferredoxin reductase FDR2 (55 kDa) in *S. peucetius* 27952. So, pEAX1/RIL which could co-express FDX1 and DoxA, AX1R2/RIL which could co-express FDX1, FDR2 and DoxA were constructed in this study, and CO-binding assay for the mixed protein was detected (Figs. 3C, 3D). The mixed protein DoxA and FDX1, and mixed protein DoxA, FDX1 and FDR2 were added with reducing agent sodium dithionite and CO was introduced, the absorption peak obviously shifted from 420 nm to 450 nm (Figs. 3C, 3D). The result showed that the active P450 forms of the mixed protein (DoxA, FDX1 and FDR2) were significantly more than that of Rimal et al. (2015), and the highest P450 activity of DoxA was seen at pEAX1/RIL (Table 3). The CO-bound reduced difference spectra of mixed proteins showed the characteristic peak at 450 nm, confirming the expression of functional P450 enzymes. It is suggested that DoxA can be folded correctly with the help of FDX1 and FDR2 to ensure its activity.

**Table 3:** The concentration of active P450 in different strains.

	22bA/RIL	28aA/RIL	pEAX1/RIL	AX1R2/RIL
Concentration of active P450 (µM)^a^	0.1780	0.4286	0.7967	10.7956

**Notes.**

a Concentration of active P450 is calculated from the reduced-CO absorbance as described in Materials and Methods.

### The determination of heme protein concentration for DoxA

**Figure 4:**
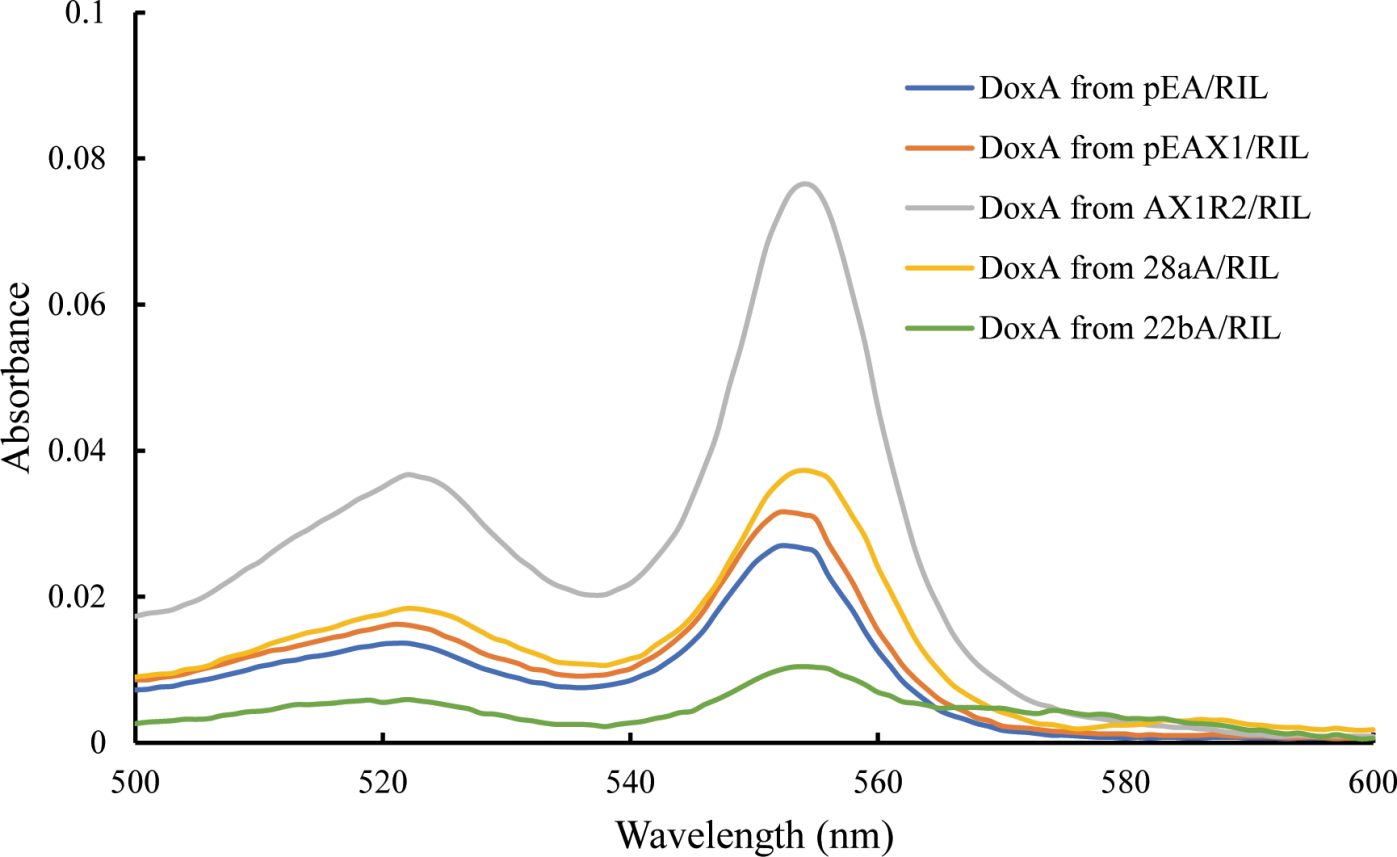
Determination for the heme concentration of DoxA in different expression strains.

The catalytic activity of P450s requires one or more redox partners to transfer two electrons from NAD(P)H to the heme iron. As a heme-containing protein, the heme concentration is also an important factor to detect the folding of DoxA. According to Barr & Guo (2015), the heme concentration of DoxA expressed in different vectors were detected, and the concentration of DoxA and mixed protein from different plasmids is keep the same. We observed the heme concentration of DoxA expressed with FDX1 and FDR2 was higher than that expressed with FDX1 and expressed alone (Fig. 4), which indicated the activity of DoxA can be enhanced with the help of FDX1 and FDR2.

### Isolation of DoxA for the co-expression strain AX1R2/RIL

**Figure 5:**
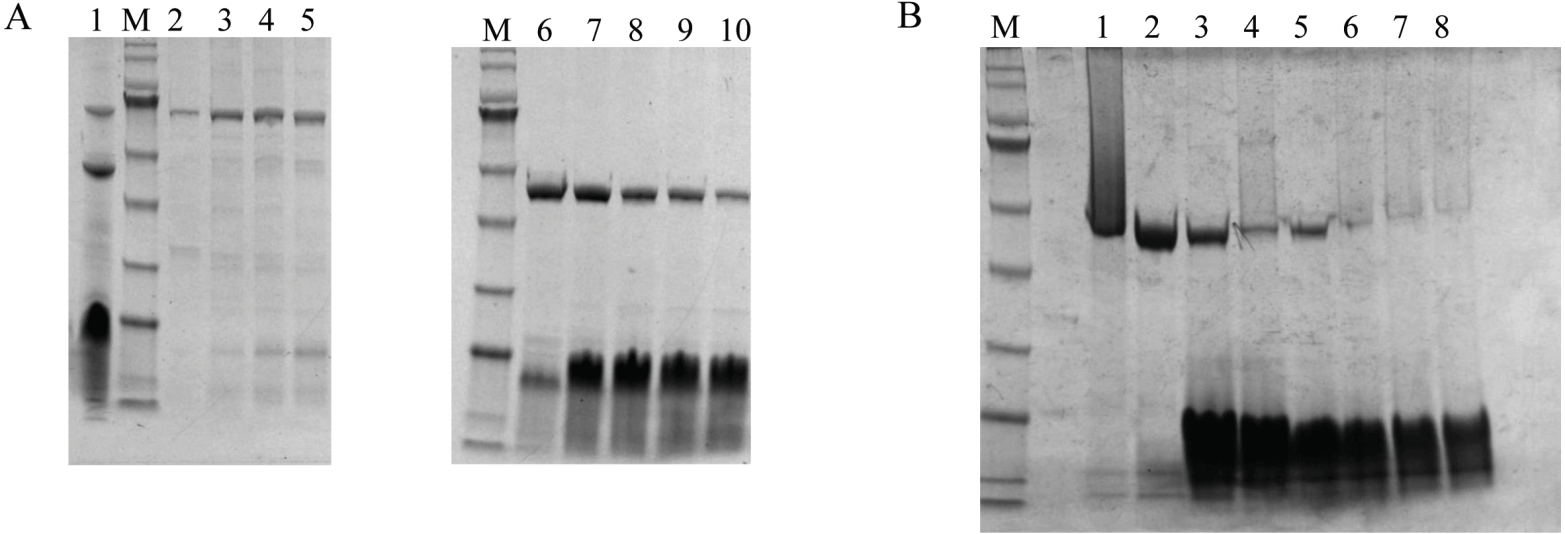
The purification and isolation of DoxA. (A), SDS-PAGE for the elution of size-exclusion chromatography. Lane 1, DoxA, FDX1 and FDR2 were purified together by Ni-NTA Resin; Lane 2–5, FDR2 was isolated by size-exclusion chromatography; Lane 6–10, DoxA and FDX1 were eluted together by size-exclusion chromatography; (B), SDS-PAGE for the elution of ion exchange chromatography. Lane 1–2, DoxA was isolated by ion exchange chromatography; Lane 7–8, FDX1 was isolated by ion exchange chromatography; M, protein marker (Genestar, 10, 15, 25, 35, 45, 65, 75, 100, 135 and 180 kDa).

DoxA, FDX1 and FDR2 were successfully purified together by Ni-NTA Resin (Fig. 5B, Lane 1), and the protein DoxA was further isolated by size-exclusion chromatography and ion exchange chromatography (buffer A: 20mM MES, 20% glycerol, pH 6.5; buffer B: 20 mM MES, 1M NaCl, 20% glycerol, pH 6.5) (Fig. 5). The result showed FDX1 was isolated by ion exchange chromatography, and FDR2 could be isolated by size-exclusion chromatography. As a result of cell culture 2.7 g/L of wet weight cell was obtained and DoxA was purified with a yield of 0.725% by Ni-NTA, 28.12% by size-exclusion chromatography, and 19.48% by ion exchange chromatography (Table 4).

### The bioconversion of DNR to DXR by DoxA

**Figure 6:**
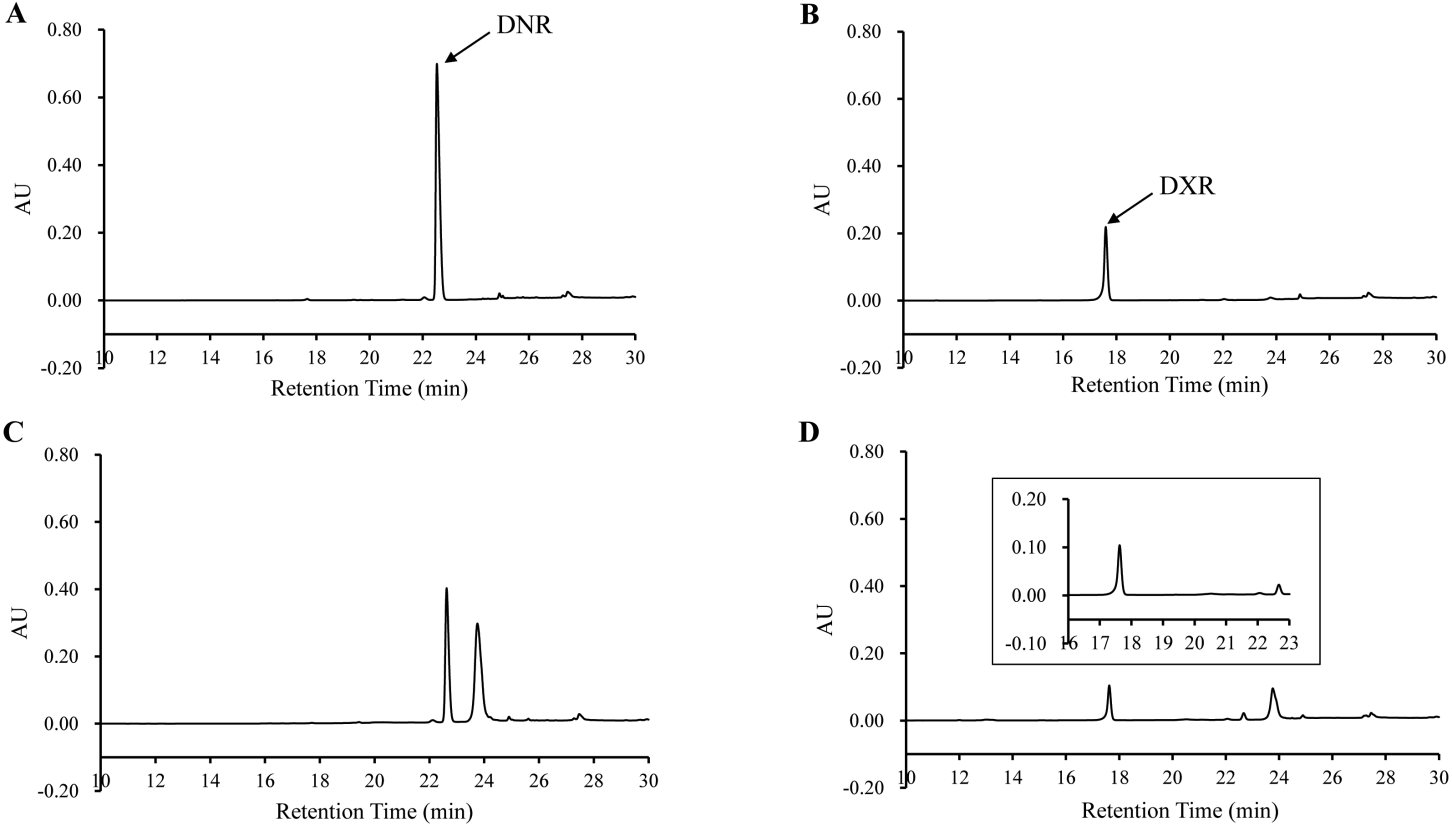
HPLC chromatograph of DoxA reactions. The peaks denote the DXR production. (A), DNR standard (100 µM); (B), DXR standard (100 µM) ; (C), Reaction control; D, Reaction of DoxA with FDX1-FDR2. The reaction mixture consisted of 6 mg mixed protein (DoxA+FDX1+FDR2), 50 µM glucose-6-phosphate, 0.5 U glucose-6-phosphate dehydrogenase, 200 µM cysteine, 5 µM NADPH, 5mM MgCl_2_ and 100 µM DNR, and the reaction was carried out in 20 mM sodium phosphate buffer (pH 7.5). The reaction mixtures were incubated at 30 °C for 24 h. While the control reaction does not contain proteins.

In order to determine the activity of DoxA, the DNR was used as substrate and the reaction product was analyzed by HPLC. The product was eluted with the same retention time (17.60 min) as authentic DXR, while there was no corresponding peak in the control (Fig. 6), which suggests DNR could be converted to DXR by DoxA using the redox partner FDX1/FDR2.

## Discussion

**Figure 7:**
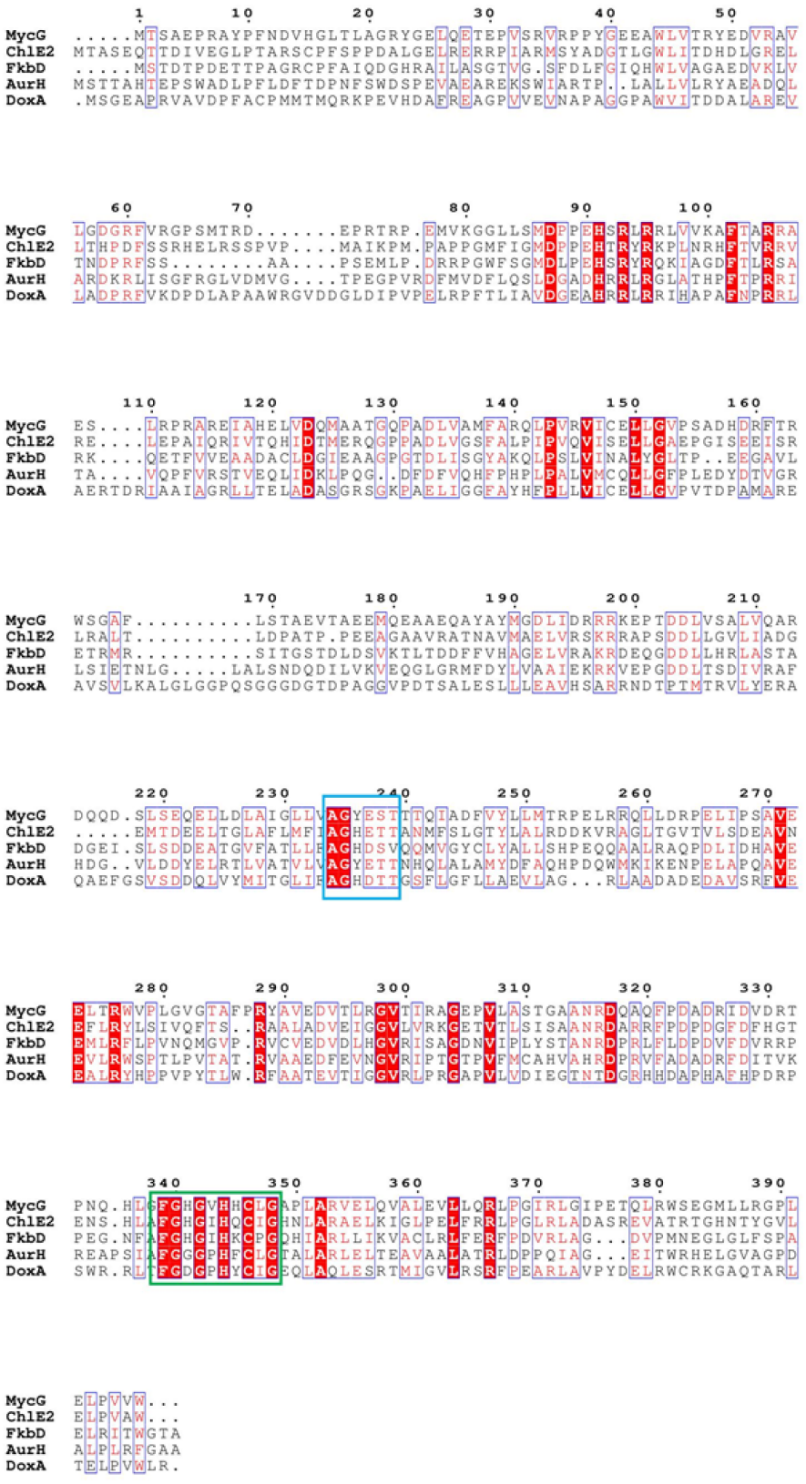
The homologous sequence alignment of DoxA. The blue box is the O_2_ binding site of P450 protein, and the green box is the iron porphyrin binding site.

DoxA belongs to CYP129A family, which contains only three proteins: DoxA from *S. peucetius* 27952, DoxA from *S. peucetius* 29050 and DoxA from *S. peucetius* C5. DoxA can catalyze continuous multi-step oxidation reactions on different carbon atoms, and the number of this kind of P450 oxidase is relatively few. The P450 oxidase MycG (PDB: 2YCA) involved in the biosynthesis of mycinamicin catalyzes hydroxylation and also epoxidation at C-14 and C-12/13 on the macrolactone ring of mycinamicin (Anzai et al., 2012; Li et al., 2012). Aurh (PDB: 3P3Z) involved in the biosynthesis of aureothin catalyzes the hydroxylation and oxidation to form the aureothin tetrahydrofuran ring (He, Müller & Hertweck, 2004; Zocher et al., 2011). Chle2 involved in chlorotricin biosynthesis can catalyze the multi-step oxidation of methyl groups at the same position of the substrate through hydroxyl, aldehyde and carboxyl groups (Jia et al., 2006). A cytochrome P450 protein, FkbD, catalyzes a less common, four-electron oxidation at C-9 to give a rarely found *α*-keto amide group (Chen et al., 2013). Sequence alignment with these P450 enzymes showed that there is highly conserved heme bound Cys, EXXR motif in K-helix and Thr in I-helix in DoxA. There are also conserved iron porphyrin binding site F-(SGNH)-X-(GD)-X-(RHPT)-X-C-(LIVMFAP)-(GAD) and O_2_ binding site (GA)-G-X-(DE)-T (Fig. 7).

**Table 4:** Summary of the DoxA purification.

Step	Total protein (mg)^a^	Target protein (mg)^b^	Purity (%)^c^
Ni-NTA	6734.6	48.9^*^	0.725
Size-exclusion chromatography	50.9	14.3	28.12
Ion exchange chromatography	7.9	1.5	19.48

**Notes.**

a Protein concentration determined by Bradford assay using BSA as a standard protein.

b Determined from total protein concentration and purity.

c Purity determined by densitometric assessment of SDS-PAGE.

* Recombinant protein.

The cytochrome *doxA* genes have been expressed previously in *E. coli*, and it is mostly in inactive P420 forms (Fig. S1, *Rimal et al., 2015*). Therefore, we attempted to develop an efficient expression system for DoxA to allow production of active, correctly folded enzyme. We firstly tried different expression vector to express DoxA alone, but the 450 nm reduced-CO difference spectrum was unable to observed (Fig. 3). This may be due to the improper protein folding or improper incorporation of the heme group into the apoenzyme in *E. coli*, just as the cytochrome P450 TxtC studied by (Healy et al., 2002).

Most CYPs require redox partner proteins to sequentially transfer two electrons form NAD(P)H to their heme–iron reactive center for dioxygen activation (Sun et al., 2017). Unlike monotonic eukaryotic cytochrome P450 reductases, bacterial redox partner systems are more diverse and complicated (Li, Du & Bernhardt, 2020). Although various orthologs of FDX and FDR are present in other bacterial strains, the heterologous reconstruction of the electron-transport partners in other host systems is often ineffective, suggesting that the employment of the most appropriate electron-transport partners is critical to obtain high CYP activity. According to Hussain & Ward (2003), we co-expressed DoxA, ferredoxin and ferredoxin reductase. CO-reduced difference spectra and heme concentration showed that DoxA could be folded correctly when co-expressed with FDX and FDR, indicating that DoxA requires the presence of redox partners to perform its function normally. It appears that co-expressing the ferredoxin reductase with P450 and ferredoxin could stabilize the folded, active form of the P450. It may suggest that an *in vivo* association of these proteins can stabilize the P450 (Hussain & Ward, 2003). It could be applied to the enhancement of other cloned P450 enzymes.

In view of the highest concentration of active p450 in the mixed protein purified from the pEAX1/RIL strain, we further isolated and purified DoxA in it. Size-exclusion chromatography is a partition chromatography that separates molecules according to their molecular sizes, so, we first used size-exclusion chromatography to separate DoxA, FDX1 and FDR2. The result showed FDR2 could be isolated, while DoxA and FDX1 could not be separated by size-exclusion chromatography (Fig. 5). This made us try ion exchange chromatography to further separate DoxA and FDX1, which is a method to separate proteins according to the different charges of proteins under certain pH conditions. The protein DoxA was finally isolated by ion exchange chromatography. Recently, our group tried to crystallize DoxA and analyze its structure for a better understanding of the protein, which can be helpful in the study of similar types of CYP monooxygenases.

The efficiency of the electron transport pathway in a bacterial Class I P450 system is determined by mutual interactions of five elements including P450, Fdx, FdR, substrate, and NADPH (Zhang et al., 2018). It is known that different redox partners can change the product distribution of a P450-catalyzed reaction (Guo et al., 2021). There are six FDXs and seven FDRs in * S. peucetius* 27952 (Rimal et al., 2015), FDX1-FDR2 was used as the redox partner of DoxA for enzyme activity experiment in our study, and the main product DXR was generated in the reaction (Fig. 6, Fig. S4). We also carried out reaction with other redox partners including 0978FDR/1499FDX (Zhang et al., 2018) and spinach FDR/FDX, and DXR was not detected using HPLC, but there were other products in these reactions (Fig. S5), which will be studied later.

## Conclusion

Overall, our work has demonstrated that it is possible to optimize construct design and expression system to generate the soluble and active DoxA. These approaches should be applicable to other P450s. The success in DoxA expression and purification will facilitate future structural studies to understand how DoxA carries out the final hydroxylation for conversion of DNR to DXR, and to reveal the molecular basis of the catalysis and exquisite substrate specificity of DoxA.

##  Supplemental Information

10.7717/peerj.14373/supp-1 Supplemental Information 1 Raw data of CO-reduced difference spectra

10.7717/peerj.14373/supp-2 Supplemental Information 2 Raw data of heme concentration

10.7717/peerj.14373/supp-3 Supplemental Information 3 CO-binding spectra of DoxA (*(Rimal et al., 2015)*) The dotted line denotes the oxidized form, and solid line denotes reduced form.

10.7717/peerj.14373/supp-4 Supplemental Information 4 The construction scheme for plasmid pEA and pEAX1

10.7717/peerj.14373/supp-5 Supplemental Information 5 The expressions of DoxA in pET22b and pET28a Lane 1, 22bA/RIL induced total protein; Lane 2, 22bA/RIL induced supernatant; Lane 3, 22bA/RIL effluent; Lane 4, 22bA/RIL washed with 50 mM imidazole; Lane 5, 22bA/RIL eluted with 500 mM imidazole; Lane 6, 28aA/RIL induced total protein; Lane 7, 28aA/RIL induced supernatant; Lane 8, 28aA/RIL effluent; Lane 9, 28aA/RIL washed with 50 mM imidazole; Lane 10, 28aA/RIL eluted with 500 mM imidazole; M, protein marker (Genestar, 15, 20, 35, 40, 50, 70, 100 and 150 kDa.)

10.7717/peerj.14373/supp-6 Supplemental Information 6 MS spectra of the reaction product The m/z_theoretical_ and m/z_observed_ values noted are for the parent ions [M+H]^+^.

10.7717/peerj.14373/supp-7 Supplemental Information 7 HPLC chromatograph of DoxA reactions with other redox partners (A), DNR standard; (B), DXR standard; C, Reaction control; D, Reaction of DoxA with 0978FDR/1499FDX; E, Reaction of DoxA with spinach FDR/FDX. The reaction mixture consisted of 6 mg mixed protein, 50 µM glucose-6-phosphate, 0.5 U glucose-6-phosphate dehydrogenase, 200 µM cysteine, 5 *μ*M NADPH, 5 mM MgCl_2_ and 100 µM DNR, and the reaction was carried out in 20 mM sodium phosphate buffer (pH 7.5). The reaction mixtures were incubated at 30 °C for 24 h. While the control reaction does not contain proteins.

## Additional Information and Declarations

### DNA Deposition

The following information was supplied regarding the deposition of DNA sequences: The doxA sequence is available at GenBank: CP022438.1.

### Data Availability

The following information was supplied regarding data availability: The raw data is available in the Supplementary Files.

## References

[ref-1] Anzai Y, Tsukada S, Sakai A, Masuda R, Harada C, Domeki A, Li S, Kinoshita K, Sherman DH, Kato F (2012). Function of cytochrome P450 enzymes MycCI and MycG in *Micromonospora griseorubida*, a producer of the macrolide antibiotic mycinamicin. Antimicrobial agents and chemotherapy.

[ref-2] Barr I, Guo F (2015). Pyridine hemochromagen assay for determining the concentration of heme in purified protein solutions. Bio-protocol.

[ref-3] Champion PM, Stallard BR, Wagner GC, Gunsalus IC (1982). Resonance Raman detection of an iron-sulfur bond in cytochrome P 450cam. Journal of the American Chemical Society.

[ref-4] Chen CC, Min J, Zhang L, Yang Y, Yu X, Guo RT (2021). Advanced understanding of the electron transfer pathway of cytochrome P450s. Chembiochem.

[ref-5] Chen D, Zhang L, Pang B, Chen J, Xu Z, Abe I, Liu W (2013). FK506 maturation involves a cytochrome p450 protein-catalyzed four-electron C-9 oxidation in parallel with a C-31 O-methylation. Journal of Bacteriology.

[ref-6] Collman JP, Sorrell TN (1975). Model for the carbonyl adduct of ferrous cytochrome P 450. Journal of the American Chemical Society.

[ref-7] de Montellano PRO (2015). Cytochrome P450: structure, mechanism, and biochemistry.

[ref-8] Guo J, Li F, Cheng F, Ma L, Liu X, Durairaj P, Zhang G, Tang D, Long X, Zhang W, Du L, Zhang X, Li S (2021). Bacterial biosynthetic P450 enzyme PikCD50N: a potential biocatalyst for the preparation of human drug metabolites. Journal of Organic Chemistry.

[ref-9] Hannemann F, Bichet A, Ewen KM, Bernhardt R (2007). Cytochrome P450 systems–biological variations of electron transport chains. Biochimica et Biophysica Acta.

[ref-10] Haudenschild C, Schalk M, Karp F, Croteau R (2000). Functional expression of regiospecific cytochrome P450 limonene hydroxylases from mint (Mentha spp.) in *Escherichia coli* and saccharomyces cerevisiae. Archives of Biochemistry and Biophysics.

[ref-11] He J, Müller M, Hertweck C (2004). Formation of the aureothin tetrahydrofuran ring by a bifunctional cytochrome p450 monooxygenase. Journal of the American Chemical Society.

[ref-12] Healy FG, Krasnoff SB, Wach M, Gibson DM, Loria R (2002). Involvement of a cytochrome P450 monooxygenase in thaxtomin A biosynthesis by Streptomyces acidiscabies. Journal of Bacteriology.

[ref-13] Hussain HA, Ward JM (2003). Enhanced heterologous expression of two *Streptomyces griseolus* cytochrome P450s and *Streptomyces coelicolor* ferredoxin reductase as potentially efficient hydroxylation catalysts. Applied and Environmental Microbiology.

[ref-14] Jia XY, Tian ZH, Shao L, Qu XD, Zhao QF, Tang J, Tang GL, Liu W (2006). Genetic characterization of the chlorothricin gene cluster as a model for spirotetronate antibiotic biosynthesis. Chemistry and Biology.

[ref-15] Klingenberg M (1958). Pigments of rat liver microsomes. Archives of Biochemistry and Biophysics.

[ref-16] Li S, Du L, Bernhardt R (2020). Redox partners: function modulators of bacterial P450 enzymes. Trends in Microbiology.

[ref-17] Li S, Tietz DR, Rutaganira FU, Kells PM, Anzai Y, Kato F, Pochapsky TC, Sherman DH, Podust LM (2012). Substrate recognition by the multifunctional cytochrome P450 MycG in mycinamicin hydroxylation and epoxidation reactions. Journal of Biological Chemistry.

[ref-18] Li Z, Jiang Y, Guengerich FP, Ma L, Li S, Zhang W (2020). Engineering cytochrome P450 enzyme systems for biomedical and biotechnological applications. Journal of Biological Chemistry.

[ref-19] Liu CJ, Huhman D, Sumner LW, Dixon RA (2003). Regiospecific hydroxylation of isoflavones by cytochrome p450 81E enzymes from *Medicago truncatula*. Plant Journal.

[ref-20] Omura T, Sato R (1962). A new cytochrome in liver microsomes. Journal of Biological Chemistry.

[ref-21] Omura T, Sato R (1964). The carbon monoxide binding pigment of liver microsomes. I. Evidence for its hemoprotein nature. Journal of Biological Chemistry.

[ref-22] Podust LM, Sherman DH (2012). Diversity of P450 enzymes in the biosynthesis of natural products. Natural Product Reports.

[ref-23] Rimal H, Lee SW, Lee JH, Oh TJ (2015). Understanding of real alternative redox partner of *Streptomyces peucetius* DoxA: prediction and validation using *in silico* and *in vitro* analyses. Archives of Biochemistry and Biophysics.

[ref-24] Rudolf JD, Chang CY, Ma M, Shen B (2017). Cytochromes P450 for natural product biosynthesis in *Streptomyces*: sequence, structure, and function. Natural Product Reports.

[ref-25] Ruettinger RT, Fulco AJ (1981). Epoxidation of unsaturated fatty acids by a soluble cytochrome P450-dependent system from *Bacillus megaterium*. Journal of Biological Chemistry.

[ref-26] Simgen B, Contzen J, Schwarzer R, Bernhardt R, Jung C (2000). Substrate binding to 15beta-hydroxylase (CYP106A2) probed by FT infrared spectroscopic studies of the iron ligand CO stretch vibration. Biochemical and Biophysical Research Communications.

[ref-27] Sono M, Andersson LA, Dawson JH (1982). Sulfur donor ligand binding to ferric cytochrome P-450-CAM and myoglobin. Ultraviolet–visible absorption, magnetic circular dichroism, and electron paramagnetic resonance spectroscopic investigation of the complexes. Journal of Biological Chemistry.

[ref-28] Stern JO, Peisach J (1974). A model compound study of the CO-adduct of cytochrome P-450. Journal of Biological Chemistry.

[ref-29] Sun Y, Ma L, Han D, Du L, Qi F, Zhang W, Sun J, Huang S, Kim ES, Li S (2017). *In vitro* reconstitution of the cyclosporine specific P450 hydroxylases using heterologous redox partner proteins. Journal of Industrial Microbiology and Biotechnology.

[ref-30] Sun Y, Zeng W, Benabbas A, Ye X, Denisov I, Sligar SG, Du J, Dawson JH, Champion PM (2013). Investigations of heme ligation and ligand switching in cytochromes p450 and p420. Biochemistry.

[ref-31] Zhang W, Du L, Li F, Zhang X, Qu Z, Han L, Li Z, Sun J, Qi F, Yao Q, Sun Y, Geng C, Li S (2018). Mechanistic insights into interactions between bacterial class I P450 enzymes and redox partners. ACS Catalysis.

[ref-32] Zocher G, Richter ME, Mueller U, Hertweck C (2011). Structural fine-tuning of a multifunctional cytochrome P450 monooxygenase. Journal of the American Chemical Society.

