# Supplementary figures and images for: Functional expression and purification of DoxA, a key cytochrome P450 from Streptomyces peucetius ATCC 27952
Source: PeerJ. 2022 Nov 16;10:e14373. doi: 10.7717/peerj.14373 (PMC9675340; doi:10.7717/peerj.14373)

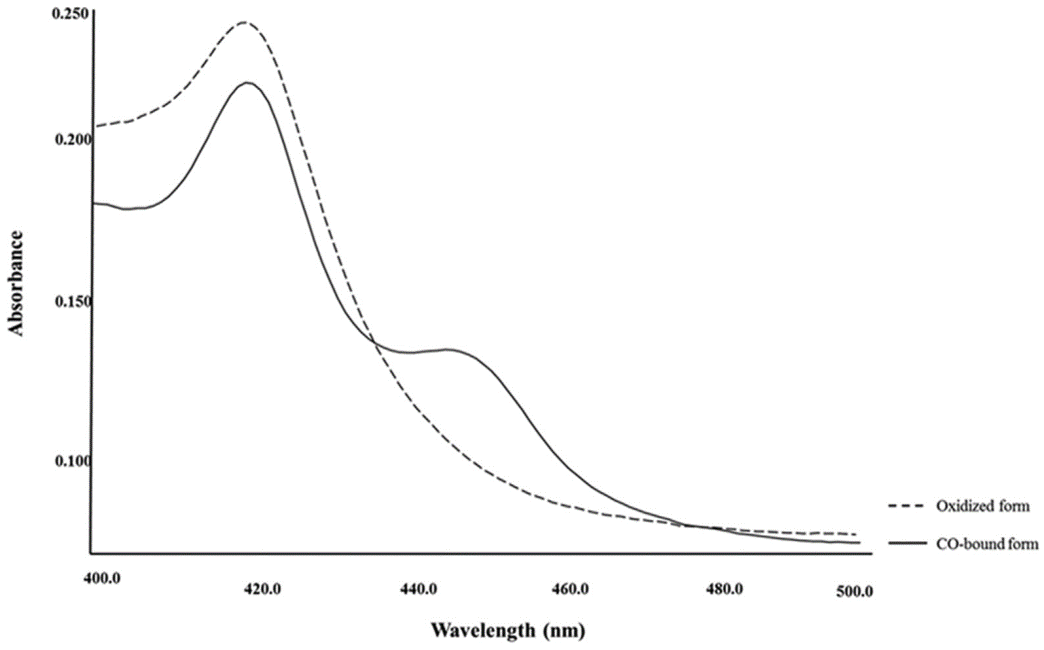

Supplement: Supplemental Information 3 — The dotted line denotes the oxidized form, and solid line denotes reduced form. [file peerj-10-14373-s003.png]

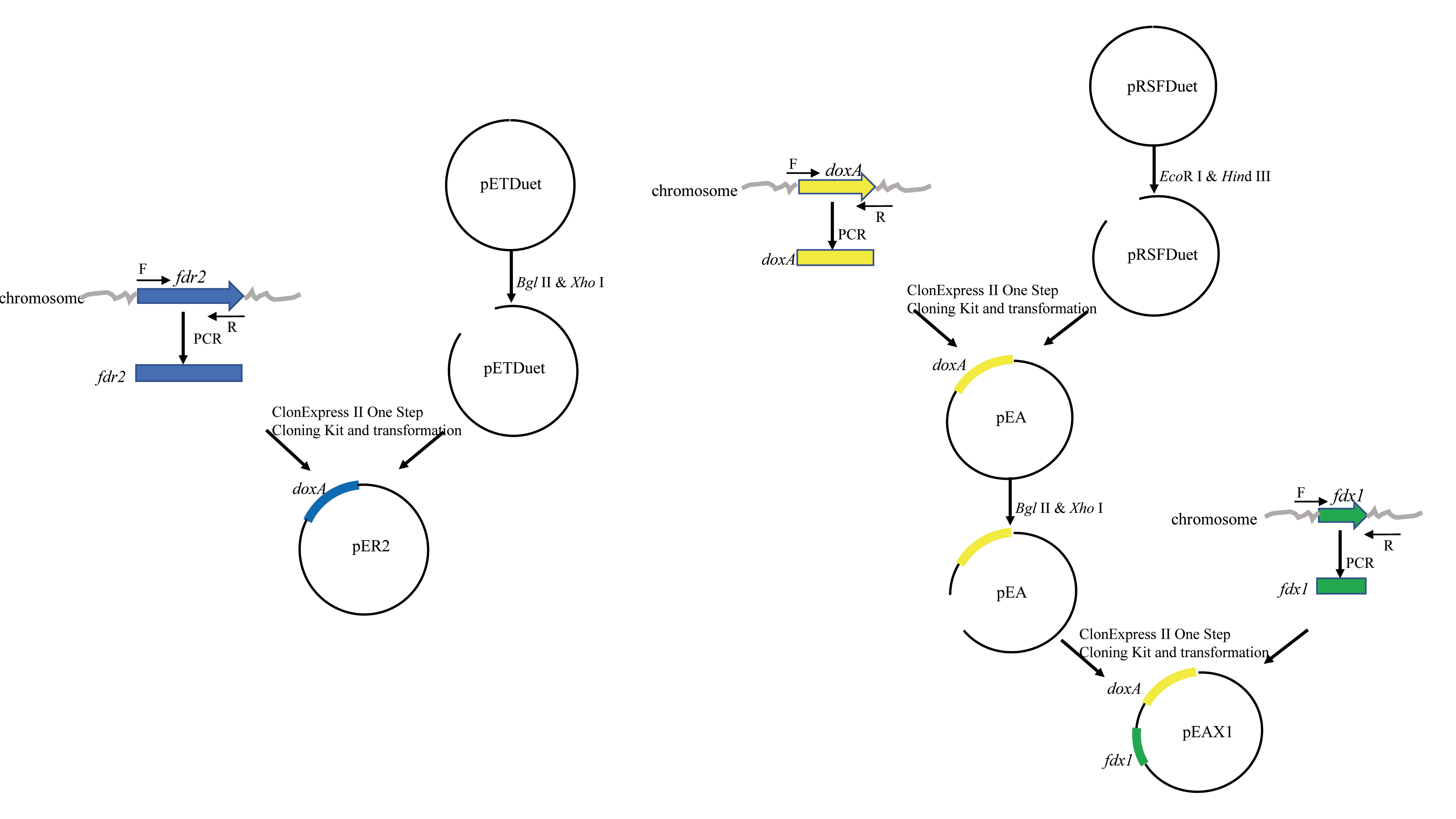

Supplement: Supplemental Information 4 [file peerj-10-14373-s004.png]

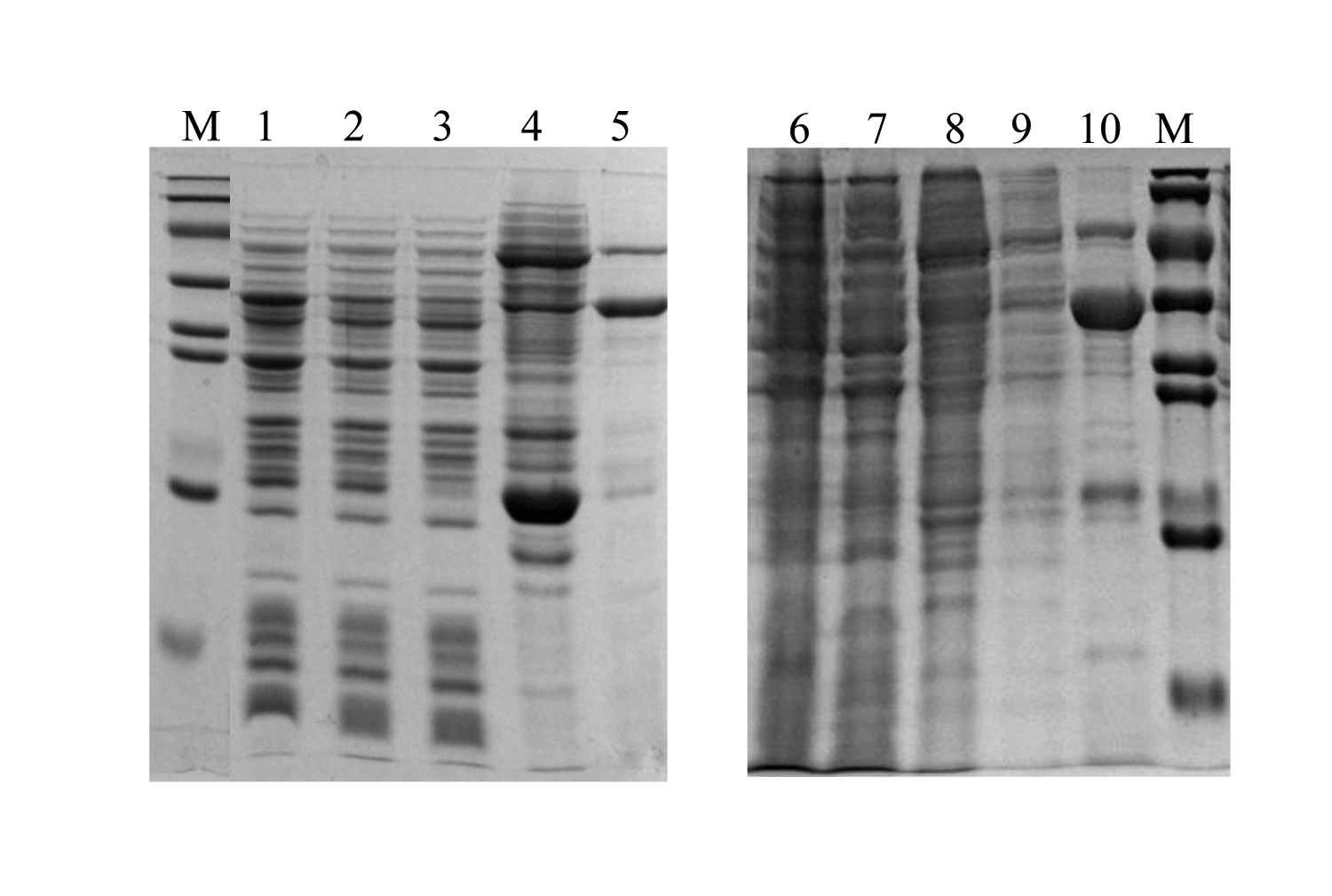

Supplement: Supplemental Information 5 — Lane 1, 22bA/RIL induced total protein; Lane 2, 22bA/RIL induced supernatant; Lane 3, 22bA/RIL effluent; Lane 4, 22bA/RIL washed with 50 mM imidazole; Lane 5, 22bA/RIL eluted with 500 mM imidazole; Lane 6, 28aA/RIL induced total protein; Lane 7, 28aA/RIL induced supernatant; Lane 8, 28aA/RIL effluent; Lane 9, 28aA/RIL washed with 50 mM imidazole; Lane 10, 28aA/RIL eluted with 500 mM imidazole; M, protein marker (Genestar, 15, 20, 35, 40, 50, 70, 100 and 150 kDa.) [file peerj-10-14373-s005.png]

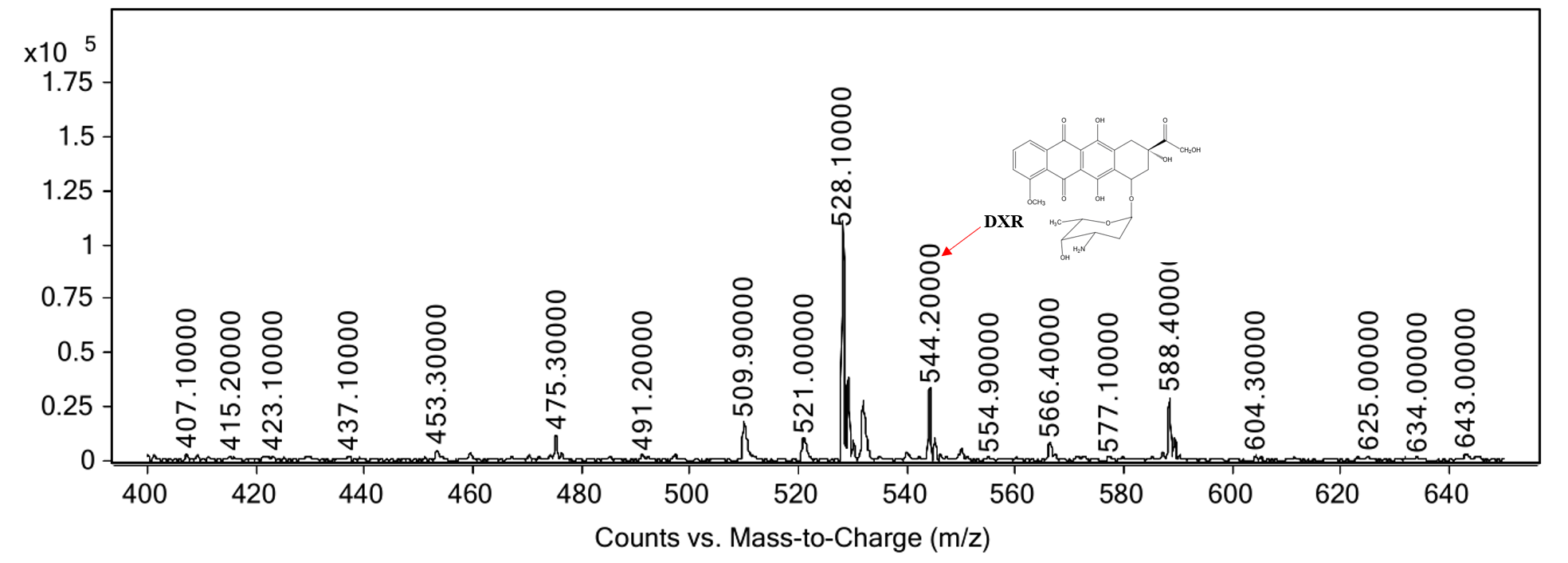

Supplement: Supplemental Information 6 — The m/ztheoretical and m/zobserved values noted are for the parent ions [M+H]+. [file peerj-10-14373-s006.png]

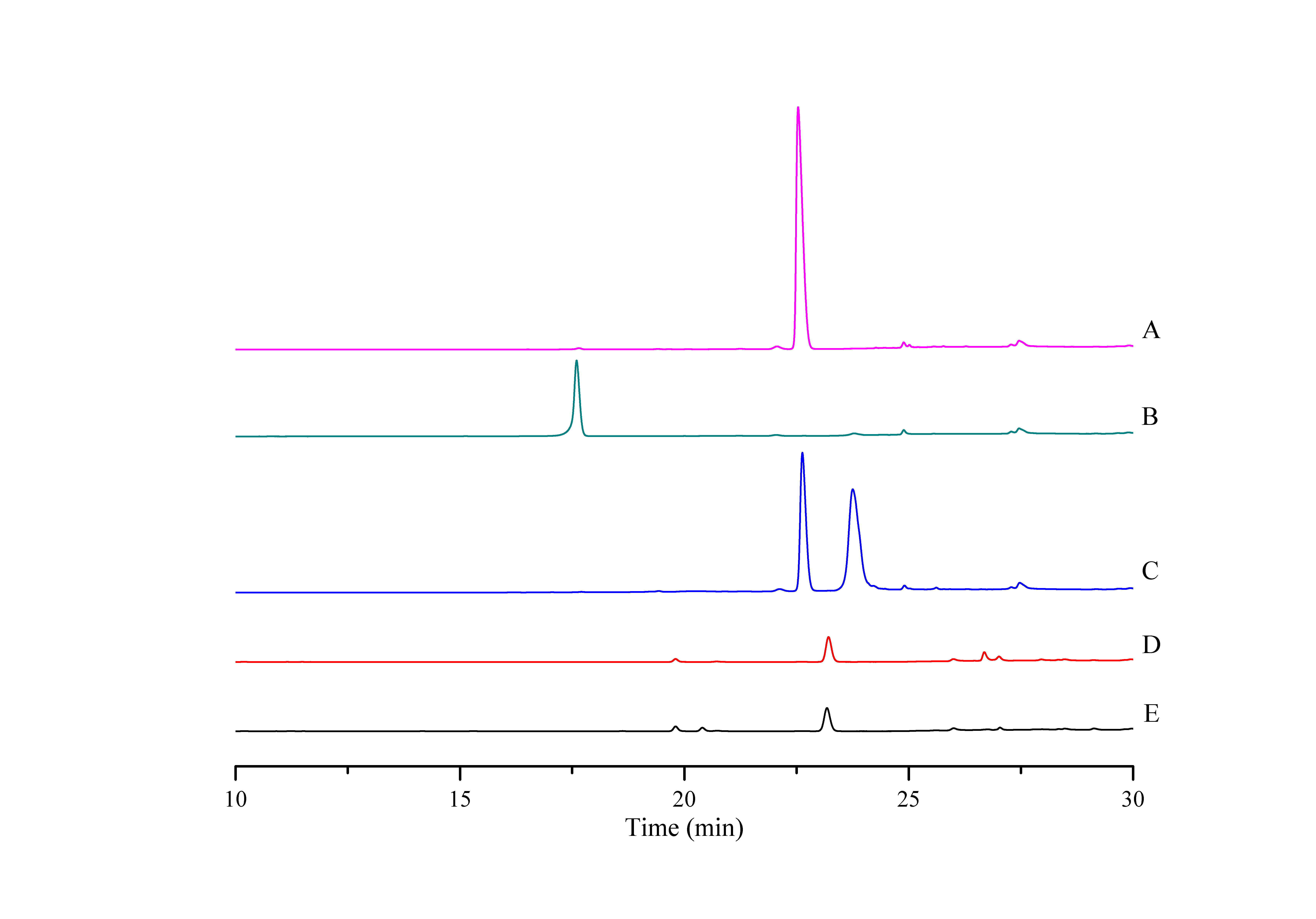

Supplement: Supplemental Information 7 — (A), DNR standard; (B), DXR standard; C, Reaction control; D, Reaction of DoxA with 0978FDR/1499FDX; E, Reaction of DoxA with spinach FDR/FDX. The reaction mixture consisted of 6 mg mixed protein, 50 µM glucose-6-phosphate, 0.5 U glucose-6-phosphate dehydrogenase, 200 µM cysteine, 5 μM NADPH, 5 mM MgCl2 and 100 µM DNR, and the reaction was carried out in 20 mM sodium phosphate buffer (pH 7.5). The reaction mixtures were incubated at 30 °C for 24 h. While the control reaction does not contain proteins. [file peerj-10-14373-s007.png]
